# The CDK9–cyclin T1 complex mediates saturated fatty acid–induced vascular calcification by inducing expression of the transcription factor CHOP

**DOI:** 10.1074/jbc.RA118.004706

**Published:** 2018-09-12

**Authors:** Yuji Shiozaki, Kayo Okamura, Shohei Kohno, Audrey L. Keenan, Kristina Williams, Xiaoyun Zhao, Wallace S. Chick, Shinobu Miyazaki-Anzai, Makoto Miyazaki

**Affiliations:** From the ‡ Division of Renal Diseases and Hypertension, Department of Medicine, and; the § Department of Cell and Developmental Biology, University of Colorado Denver, Aurora, Colorado 80045

**Keywords:** endoplasmic reticulum stress (ER stress), vascular smooth muscle cells, fatty acid, cyclin-dependent kinase (CDK), vascular biology, CDK9, CHOP, ER stress, saturated fatty acids, vascular calcification, kidney disease, renal dysfunction, atherosclerosis, cardiovascular disease

## Abstract

Vascular calcification (or mineralization) is a common complication of chronic kidney disease (CKD) and is closely associated with increased mortality and morbidity rates. We recently reported that activation of the activating transcription factor 4 (ATF4) pathway through the saturated fatty acid (SFA)-induced endoplasmic reticulum (ER) stress response plays a causative role in CKD-associated vascular calcification. Here, using mouse models of CKD, we 1) studied the contribution of the proapoptotic transcription factor CCAAT enhancer–binding protein homologous protein (CHOP) to CKD-dependent medial calcification, and 2) we identified an additional regulator of ER stress–mediated CHOP expression. Transgenic mice having smooth muscle cell (SMC)–specific CHOP expression developed severe vascular apoptosis and medial calcification under CKD. Screening of a protein kinase inhibitor library identified 16 compounds, including seven cyclin-dependent kinase (CDK) inhibitors, that significantly suppressed CHOP induction during ER stress. Moreover, selective CDK9 inhibitors and CRISPR/Cas9-mediated CDK9 reduction blocked SFA-mediated induction of CHOP expression, whereas inhibitors of other CDK isoforms did not. Cyclin T1 knockout inhibited SFA-mediated induction of CHOP and mineralization, whereas deletion of cyclin T2 and cyclin K promoted CHOP expression levels and mineralization. Of note, the CDK9–cyclin T1 complex directly phosphorylated and activated ATF4. These results demonstrate that the CDK9–cyclin T1 and CDK9–cyclin T2/K complexes have opposing roles in CHOP expression and CKD-induced vascular calcification. They further reveal that the CDK9–cyclin T1 complex mediates vascular calcification through CHOP induction and phosphorylation-mediated ATF4 activation.

## Introduction

Medial calcification is a common complication of chronic kidney disease (CKD)^2^ and a strong predictor of mortality and morbidity (1, 2). Medial calcification is more prevalent in patients with CKD than atherosclerotic calcification. We previously reported that serum levels of total saturated fatty acids (SFA), such as palmitic acid (C16:0) and stearic acid (C18:0), are positively associated with cardiovascular calcification in patents with CKD (3). Consistently, accumulation of SFAs in vascular smooth muscle cells (VSMCs) due to the inhibition of stearoyl-CoA desaturase induced severe medical calcification in mice. Recent evidence suggests that endoplasmic reticulum (ER) stress is a major contributor to the pathogenesis of vascular and valvular calcification in CKD (2–11). The ER is a major site of calcium regulation, protein folding, and lipid metabolism (12). ER stress is a defense system activated to maintain ER homeostasis and is initiated by calcium imbalance and accumulation of unfolded proteins and SFAs. The three ER resident sensors, PKR-like endoplasmic reticulum kinase (PERK), inositol-requiring enzyme 1 (IRE1), and activating transcription factor 6 (ATF6), implement the ER stress response (13). We previously reported that the PERK-initiated pathway of the ER stress response is critical in the mineralization of VSMCs (3, 8–11). Activating transcription factor 4 (ATF4) is a pivotal transcription factor in the PERK–eukaryotic protein initiation factor 2α (eIF2α) pathway of the ER stress response and in osteoblast differentiation (14–17). ATF4 targets CHOP, resulting in apoptosis under ER stress (4, 12, 18). We recently reported that CKD induced levels of aortic ER stress markers such as ATF4 and CHOP due to accumulation of aortic SFAs such as stearic acid (3, 10, 11). Global haploinsufficient and smooth muscle cell (SMC)-specific knockout of ATF4 attenuated both medial and atherosclerotic calcification in mouse models of CKD (11).

Cyclin-dependent kinases (CDKs) are members of the serine/threonine kinase subfamily (19–21). Cyclins are regulatory subunits that bind to the CDK, resulting in activation of the kinase. Most members of the CDK family form a CDK–cyclin complex and are involved in the regulation of either cell cycle or transcription. The human genome encodes 21 CDKs (1–11a and 11b-20) and over 15 cyclins (A–L, O, T, and Y) (19–22). CDK9 does not act in cell-cycle regulation processes; rather, it acts in differentiation processes (22–30). CDK9 forms heterodimeric complexes with cyclins T1, T2a, T2b, and K (22, 31–34). Cyclins T2a and T2b are splice variants with T2b having an additional 67 amino acids at its C terminus. CDK9 is expressed ubiquitously in all tissues, as are its activators, cyclins T1, T2a, and T2b (22, 31, 33). CDK9–cyclin heterodimers are components of a larger protein complex called positive transcription elongation factor b (P-TEFb). CDK9 in the P-TEFb complex phosphorylates the C-terminal domain of RNA polymerase II, a key regulatory mechanism during elongation (35–38). *In vitro*, all four complexes (CDK9–cyclin T1, CDK9–cyclin T2a, CDK9–cyclin T2b, and CDK9–cyclin K) possess transcription elongation activity (33, 34). The CDK9–cyclin T2 complex phosphorylates myoblast determination protein 1 to regulate muscle differentiation (27, 29). However, which CDK9–cyclin complex contributes to ER stress-induced CHOP expression and medial calcification has yet to be determined.

In this context, we examined whether CHOP induction solely induces medial calcification in CKD, and we identified a novel pathway that contributes to ER stress-induced CHOP expression and vascular calcification.

## Results

### SMC-specific CHOP overexpression augments medial calcification in CKD

**Figure 1.**
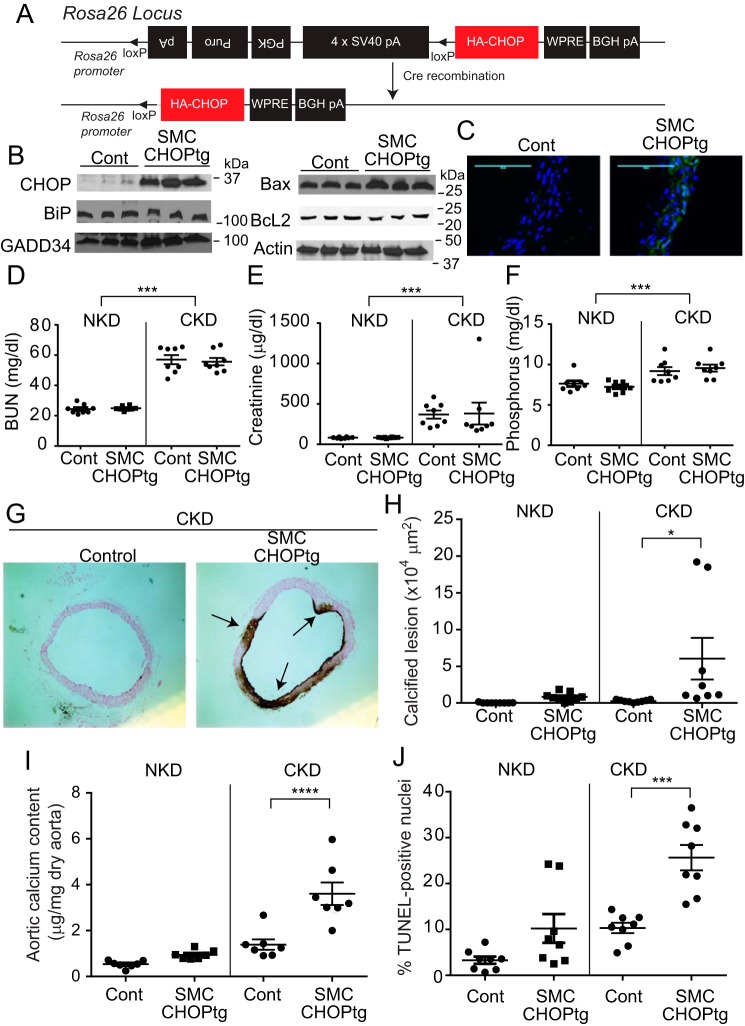
**SMC-specific CHOP overexpression induces medial calcification.** *A,* scheme of construct design for targeting the CHOP transgene in the Rosa26 locus. *Triangles* indicate the loxP site. CHOP conditional TG mice were backcrossed 10 times with C57Bl6 mice. CHOP conditional TG mice were crossed with SMMHC–Cre^(ER)T2^ TG mice to generate SMC–CHOP TG mice. 5-Week-old male mice under NKD were injected with vehicle or 1 mg of tamoxifen for 5 consecutive days to generate control mice and SMC–CHOP TG mice, respectively. The 8-week-old mice were subjected to either sham operation (*NKD*) or 5/6 nephrectomy (*CKD*) and were sacrificed at 20 weeks of age. *B,* immunoblot analysis of CHOP and its targets (BiP, GADD34, Bax, and BcL2) in the aortic media. The medial layer of aortas was isolated from control and SMC–CHOP TG mice. *C,* immunofluorescence microscopic analysis of CHOP (*green*) in the aortic arch. DAPI (*blue*). *D–F*, levels of BUN, serum creatinine, and phosphorus in SMC–CHOP TG mice. Serum creatinine levels were analyzed by LC-MS/MS. Other parameters were analyzed with colorimetric assays. *G,* representative photograph (×10) of the lesions in aortic arches stained with von Kossa. *Arrows* (black lesions) indicate calcification. *H,* quantitative analysis of calcified lesions in the aortic arches. *I,* aortic calcium content in SMC–CHOP TG mice. *J,* quantitative analysis of apoptotic lesions (terminal deoxynucleotidyltransferase-mediated dUTP nick end labeling (TUNEL)–positive nuclei) in the aortic arches of SMC–CHOP TG mice. Two-way ANOVA was used for comparison between NKD and CKD. *n* = 8; *, *p* < 0.05; ***, *p* < 0.001; and ****, *p* < 0.0001; *Cont,* control.

To study the SMC-specific role of CHOP in regulating medial calcification, we generated CHOP conditional transgenic (loxTG) mice similar to the ATF4 loxTG mice that we previously reported (11). The random insertion of a transgene into the mouse genome may cause deregulation or misregulation of the expression of essential genes. To overcome this issue, a conditional CHOP transgene was inserted to a known permissive locus, Rosa26, by a recombinase-mediated cassette exchange procedure (Fig. 1*A*). Conditional CHOP transgenic (Rosa26–CHOP^loxtg/+^) mice were bred with SMMHC–CreER^(T2)^ mice to generate SMC–CHOP conditional transgenic (SMMHC–CreER^(T2)^; Rosa–CHOP^loxtg/+^) mice. Injection of tamoxifen but not vehicle in SMC–CHOP conditional transgenic mice caused CHOP overexpression (Fig. 1, *B* and *C*). Tamoxifen injection induced the expression of the CHOP transgene in SMCs but not in fibroblasts, macrophages, neutrophils, hepatocytes, the brain, or skeletal muscles (data not shown). SMC–CHOP overexpression affected target gene expression. Protein levels of Bax and GADD34 were increased in the aortic media of SMC–CHOP TG mice, whereas BcL2 and BiP protein levels were reduced (Fig. 1*B*). As expected, CKD increased levels of BUN, serum creatinine, and phosphorus, which were not altered by SMC–CHOP overexpression (Fig. 1, *D–F*). Under normal kidney conditions (NKD), control WT and SMC–CHOP TG mice did not show calcified lesions in the aortic arches, whereas SMC–CHOP TG mice, but not control mice under CKD, displayed severe medial calcification (Fig. 1, *G* and *H*). In addition, aortic calcium content and vascular apoptosis were increased by 2.7- and 2.3-fold, respectively, in SMC–CHOP TG mice compared with control mice under CKD. Under the NKD condition, there was no significant different in aortic calcium content and vascular apoptosis (Fig. 1, *I* and *J*).

### Inhibition of CDK9 blocks SFA-induced CHOP expression

**Figure 2.**
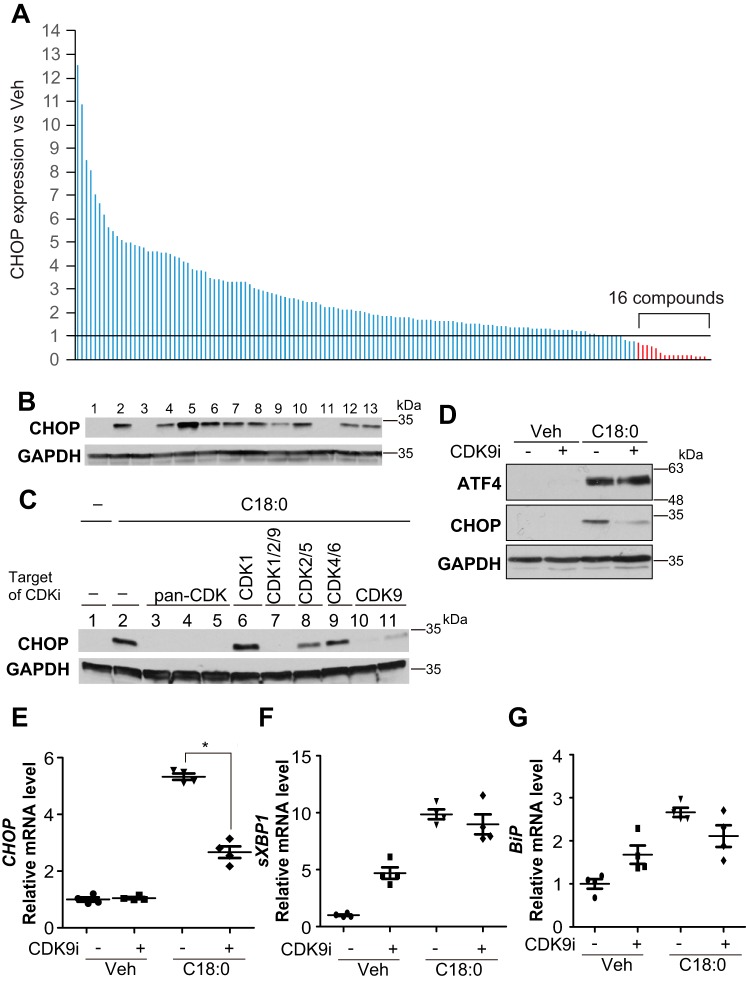
**CDK9 regulates CHOP expression.** *A,* immunoblot analysis of CHOP protein to screen for inhibition of SFA-induced CHOP induction with a kinase inhibitor library containing >140 compounds. VSMCs were pretreated with 10 μm of each inhibitor for 2 h prior to co-treatment with 500 μm C18:0 and 10 μm of each inhibitor for 6 h. CHOP expression is expressed as a fold value relative to that of vehicle-treated (DMSO-treated, fold value is 1) cells with GAPDH correction by band densitometry. The experiments were repeated three times. *B,* immunoblot analysis of CHOP protein to screen for inhibition of SFA-induced CHOP induction. VSMCs were pretreated with 10 μm of each inhibitor for 2 h prior to co-treatment with 500 μm C18:0 and 10 μm of each inhibitor for 6 h. Total cell lysates of VSMCs were then prepared. BSA (*Veh*) with DMSO (*lane 1*), C18:0 with DMSO (*lane 2*), C18:0 with triacin C (acyl-CoA–specific synthetase inhibitor, *lane 3*), C18:0 with H89 (PKA inhibitor, *lane 4*), C18:0 with Gö6983 (PKC inhibitor, *lane 5*), C18:0 with Akti-1/2 (Akt inhibitor, *lane 6*), C18:0 with U0126 (MEK inhibitor, *lane 7*), C18:0 with rapamycin (mTOR inhibitor, *lane 8*), C18:0 with SB2003580 (p38 MAPK inhibitor, *lane 9*), C18:0 with SP600125 (JNK inhibitor, *lane 10*), C18:0 with AT7519 (pan-CDK inhibitor, *lane 11*), C18:0 with SL0101–1 (RSK inhibitor, *lane 12*), and C18:0 with CHIR99021 (GSK-3 inhibitor, *lane 13*) are as indicated. *C,* immunoblot analysis of CHOP protein to screen for CDK-specific inhibition of SFA-induced CHOP induction. VSMCs were pretreated with 10 μm of each inhibitor for 2 h prior to co-treatment with 500 μm C18:0 and 10 μm of each inhibitor for 6 h. Total cell lysates of VSMCs were then prepared. BSA (*Veh*) with DMSO (*lane 1*), C18:0 with DMSO (*lane 2*), AT7519 (*lane 3*), flavopiridol (*lane 4*), dinaciclib (*lane 5*), RO-3306 (*lane 6*), AZD5438 (*lane 7*), roscovitine (*lane 8*), palbociclib (*lane 9*), CAY10574 (*lane 10*), and LDC000067 (*lane 11*) in the presence of 500 μm C18:0 are as shown. *D,* immunoblot analysis of ATF4, CHOP, and GAPDH proteins in VSMCs co-treated with SFA and CDK9 inhibitor. VSMCs were pretreated with DMSO (−) or 30 μm CAY10574 (CDK9 inhibitor) for 2 h prior to co-treatment with 250 μm C18:0 and DMSO or 30 μm CAY10574 for 6 h. Total cell lysates of VSMCs were then prepared. qRT-PCR analyses of CHOP (*E*), *sXBP-1* (*F*), and *BiP* (*G*) in VSMCs co-treated with SFA and CDK9 inhibitor are as shown. VSMCs were pretreated with DMSO or 30 μm CAY10574 (CDK9 inhibitor) for 2 h prior to co-treatment with 250 μm C18:0 and 30 μm CAY10574 for 6 h. Total RNA of VSMCs was then prepared (*n* = 4). One-way ANOVA with a Student-Newman post hoc test was used for statistical analysis. *, *p* < 0.05.

**Figure 3.**
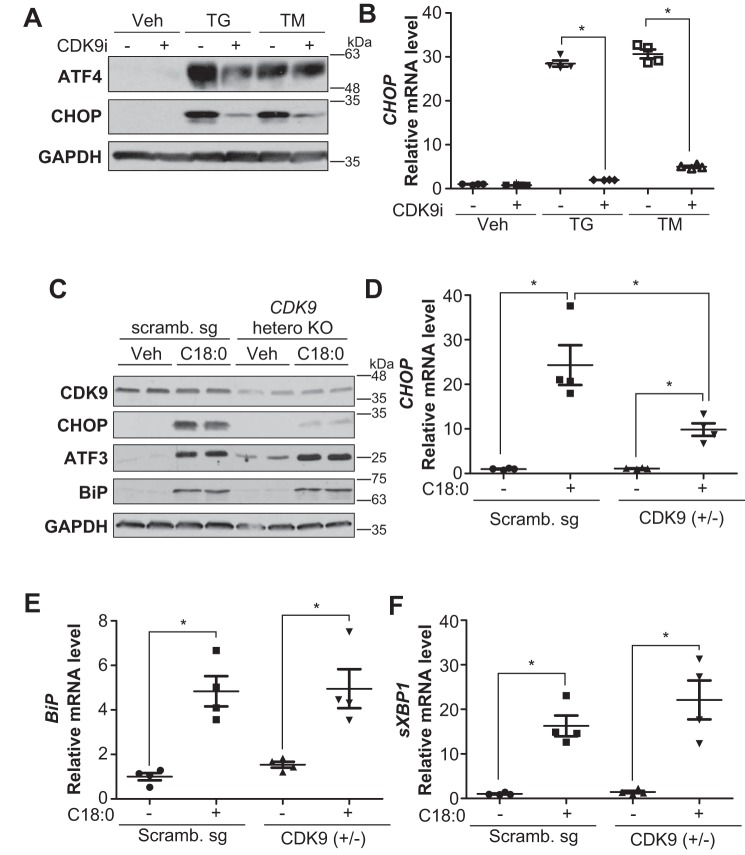
**CDK9 inhibitor or heterozygous knockout (+/−) of CDK9 blocked ER stress-mediated CHOP induction in VSMCs.** *A,* immunoblot analysis of ATF4, CHOP, and GAPDH in VSMCs co-treated with thapsigargin (*TG*) or tunicamycin (*TM*) and CDK9 inhibitor. VSMCs were pretreated with DMSO or 30 μm CAY10574 for 2 h prior to co-treatment with DMSO (*Veh*), 0.1 μm thapsigargin, or 0.1 μg/ml tunicamycin and DMSO (−) or 30 μm CAY10574 (+) for 6 h. *B,* qRT-PCR analysis of CHOP in VSMCs co-treated with thapsigargin or tunicamycin and CDK9 inhibitor. VSMCs were pretreated with DMSO or 30 μm CAY10574 for 2 h prior to treatment with DMSO (*Veh*) or co-treatment with 0.1 μm thapsigargin, or 0.1 μg/ml tunicamycin and DMSO (−) or 30 μm CAY10574 (+) for 6 h. Total RNA of VSMCs was then prepared (*n* = 4). *C,* immunoblot analysis of CDK9, CHOP, ATF3, BiP, and GAPDH proteins in BSA (*Veh*) or 500 μm C18:0 treated scrambled (*Scramb.*) sg or CDK9 heterozygous KO VSMCs for 6 h. *D,* levels of CHOP; *E, BiP*; and *F, sXBP-*1 by qRT-PCR in BSA or 250 μm C18:0 treated Scramb. sg or CDK9 hetero-KO VSMCs (*n* = 4). One-way ANOVA with a Student-Newman post hoc test was used for statistical analysis. *, *p* < 0.05.

To explore a signaling pathway that blocks SFA-induced CHOP expression, we screened a kinase inhibitor library containing >140 compounds by treating human VSMCs with a saturated fatty acid, stearic acid (C18:0). Based on the library screening, we found that 16 kinase inhibitors blocked C18:0-induced CHOP expression, seven of which were surprisingly cyclin-dependent kinase inhibitors (Fig. 2*A* and Table 1). We also confirmed that pan-CDK inhibitors but not common protein kinase inhibitors (MEK, PKA, RSK, and p38 MAPK) blocked C18:0-induced CHOP expression in mouse VSMCs, similar to human VSMCs (Fig. 2*B*) The pan-CDK inhibitors such as flavopiridol and AT7519 have potent inhibitory activity against multiple CDK isoforms. To identify which CDK isoform regulates SFA-induced CHOP expression, VSMCs were treated with several isoform-specific CDK inhibitors. Interestingly, only compounds inhibiting CDK9, including AT7519 (*lane 3*), flavopiridol (*lane 4*), dinaciclib (*lane 5*), AZD5438 (*lane 7*), CAY10574 (*lane 10*), and LDC000067 (*lane 11*) reduced levels of CHOP induced by C18:0 (Fig. 2*C*), suggesting that CDK9 regulates CHOP expression. CDK9-specific inhibitor treatment reduced levels of CHOP protein and mRNA (Fig. 2, *D* and *E*), whereas other ER stress markers such as ATF4 (Fig. 2*D*), *sXBP-1* (Fig. 2*F*), *BiP* (Fig. 2*G*), and ATF3 (data not shown) were not affected. We next confirmed whether CDK9 regulates ER stress-mediated CHOP expression using two common ER stress inducers, thapsigargin and tunicamycin. CDK9-specific inhibitor (CAY10574) blocked the induction of CHOP by thapsigargin and tunicamycin (Fig. 3, *A* and *B*). To further confirm that CDK9 contributes to ER stress-mediated CHOP expression, CDK9 knockout VSMCs were generated using a CRISPR–Cas9 system. Because CDK9 homozygous (−/−) VSMCs were not viable, CDK9 heterozygous (+/−) VSMCs were used. CDK9 haplo-insufficiency blocked C18:0-mediated CHOP but not other ER stress effectors such as *BiP* and *sXBP1*, consistent with treatment with CDK9-specific inhibitors (Fig. 3, *C–F*). These data suggest that CDK9 selectively regulates CHOP induction under ER stress.

**Table 1:** **List of inhibitors that block SFA-induced CHOP expression**

Inhibitor name		CHOP expression (ratio to C18:0 with vehicle)
Olomoucine	Cyclin-dependent kinases (CDK) inhibitor	0.63
INK128	TORC1/2 inhibitor	0.59
PI3Kα inhibitor 2	Phosphatidylinositol 3-kinase (PI3Kα) inhibitor	0.59
CAY10574	CDK9 and CDK2-cyclin E inhibitor	0.50
Torin 1	mTOR selective inhibitor	0.28
Kenpaullone	CDKs and glycogen synthase kinase 3β (GSK3β) inhibitor	0.19
5-Iodotubercidin	Nonspecific PK inhibitor	0.19
Bisindolylmaleimide IX (mesylate)	Protein kinase C (PKC) isoforms inhibitor	0.19
NSC 663284	Cdc25 isoforms inhibitor	0.18
Wortmannin	PI3K enzymes inhibitor	0.17
PHA-767491	CDK7/CDK9 kinase inhibitor	0.17
GSK1059615	PI3Kα inhibitor	0.17
BIBF 1120	Tyrosine kinase inhibitor	0.14
Dinaciclib	pan-CDK inhibitor	0.14
AT7519	pan-CDK inhibitor	0.13
Flavopiridol	pan-CDK inhibitor	0.02
C18:0 with vehicle	BSA	1.00
Vehicle	BSA	0.15

### CDK9 inhibitor blocked vascular calcification in CKD

**Figure 4.**
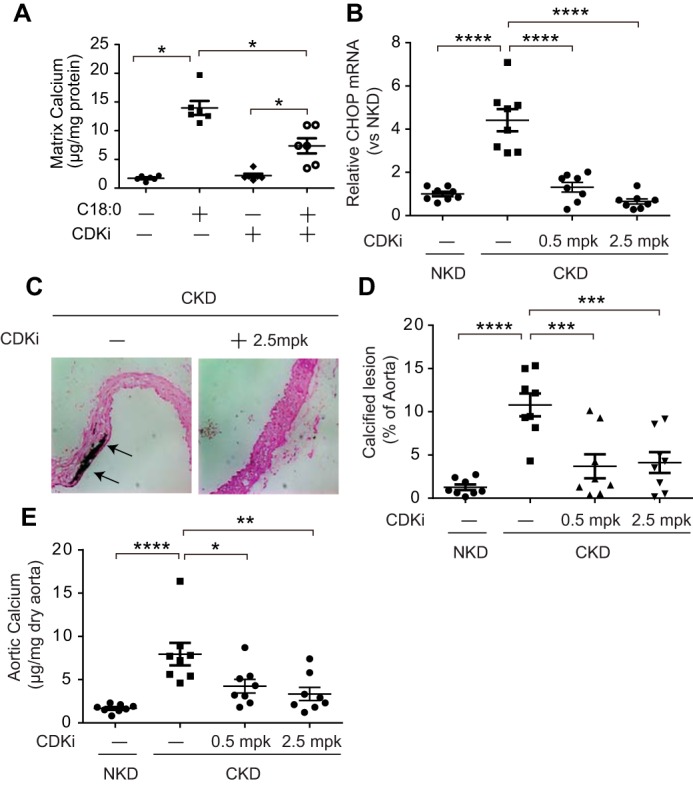
**Flavopiridol blocks vascular calcification *in vitro* and *in vivo*.** *A,* mineralization of VSMCs treated with C18:0 and CDK9 inhibitor. VSMCs were incubated with 2.0 mm phosphate with BSA and DMSO (*Veh*), 250 μm C18:0 and DMSO, or 50 nm flavopiridol (*CDKi*) for 7 days (*n* = 6). *, *p* < 0.05. One-way ANOVA with a Student-Newman post hoc test was used for comparison between DMSO and flavopiridol-treated VSMCs. *B,* aortic CHOP expression in DBA/2J mice treated with flavopiridol. 5/6 nephrectomized DBA/2J mice (*n* = 8) were treated by daily i.p. injection of flavopiridol (CDKi, 0.5 or 2.5 mg/kg) for 8 weeks. *C,* representative photograph (×10) of the lesions in aortic arches stained with von Kossa. *Arrows* (black lesions) indicate calcification. *D,* quantitative analysis of calcified lesions in the aortic arches; *E,* aortic calcium content of DBA/2J mice treated with flavopiridol. ****, *p* < 0.0001; ***, *p* < 0.001; **, *p* < 0.01; and *, *p* < 0.05 (one-way ANOVA with a Student-Newman post hoc test).

We next investigated whether CDK9 inhibition blocks mineralization of VSMCs *in vitro* and CKD-dependent vascular calcification *in vivo*. Because orally active and potent CDK9-specific inhibitor is not commercially available, we used flavopiridol, which preferentially inhibits CDK9 over other CDK isoforms at a low dose (39–41). First, we determined whether flavopiridol blocks mineralization of VSMCs. Flavopiridol treatment significantly reduced C18:0-induced mineralization of VSMCs (Fig. 4*A*). We next examined whether flavopiridol treatment prevents CKD-dependent vascular calcification *in vivo*. DBA/2J mice with 5/6 nephrectomies were treated with 0.5 or 2.5 mg/kg flavopiridol for 8 weeks. Flavopiridol treatment did not affect levels of serum creatinine, phosphorus, and calcium (data not shown). Similar to previous studies, CKD (5/6 nephrectomy) increased levels of aortic CHOP mRNA by 4.4-fold compared with NKD (sham operation) (8, 9). Both 0.5 and 2.5 mg/kg flavopiridol treatment completely reduced CKD-mediated CHOP induction (Fig. 4*B*), resulting in marked attenuation of CKD-dependent medial calcification in DBA/2J mice (Fig. 4, *C–E*).

**Figure 5.**
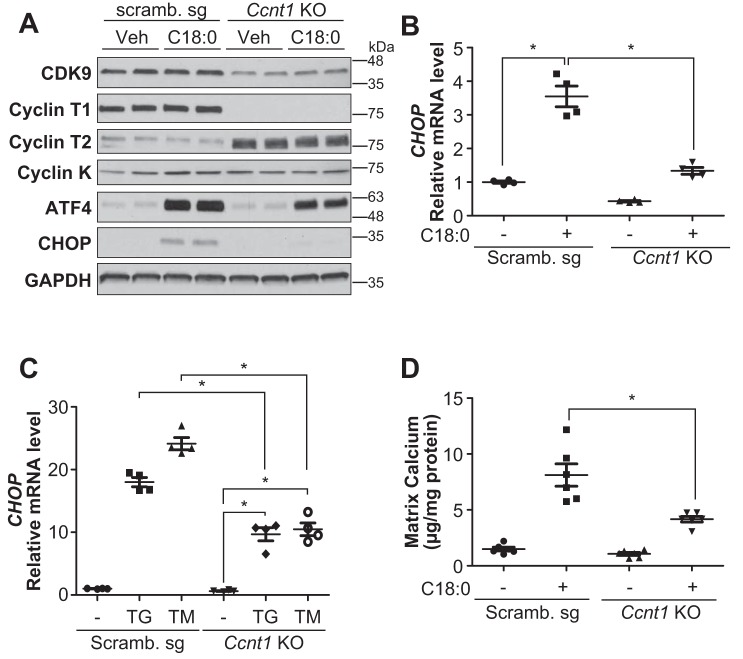
**Cyclin T1 (*Ccnt1*) gene is required for ER stress-mediated CHOP induction and vascular calcification.** *A,* immunoblot analysis of CDK9, cyclin T1, cyclin T2, cyclin K, ATF4, CHOP, and GAPDH proteins in BSA (*Veh*) or 500 μm C18:0 treated scrambled (*Scramb.*) sg or *Ccnt1* KO VSMCs for 6 h. *B,* qRT-PCR analysis of CHOP in BSA or 250 μm C18:0 treated Scramb. sg or *Ccnt1* KO VSMCs (*n* = 4). *C,* qRT-PCR analysis of CHOP in DMSO, 0.1 μm thapsigargin (*TG*), or 0.1 μg/ml tunicamycin (*TM*)-treated Scramb. sg or *Ccnt1* KO VSMCs (*n* = 4). *D,* mineralization of *Ccnt1* KO VSMCs. VSMCs were incubated with 2.0 mm phosphate with or without 250 μm C18:0 for 7 days (*n* = 6). One-way ANOVA with a Student-Newman post hoc test was used for statistical analysis. *, *p* < 0.05.

**Figure 6.**
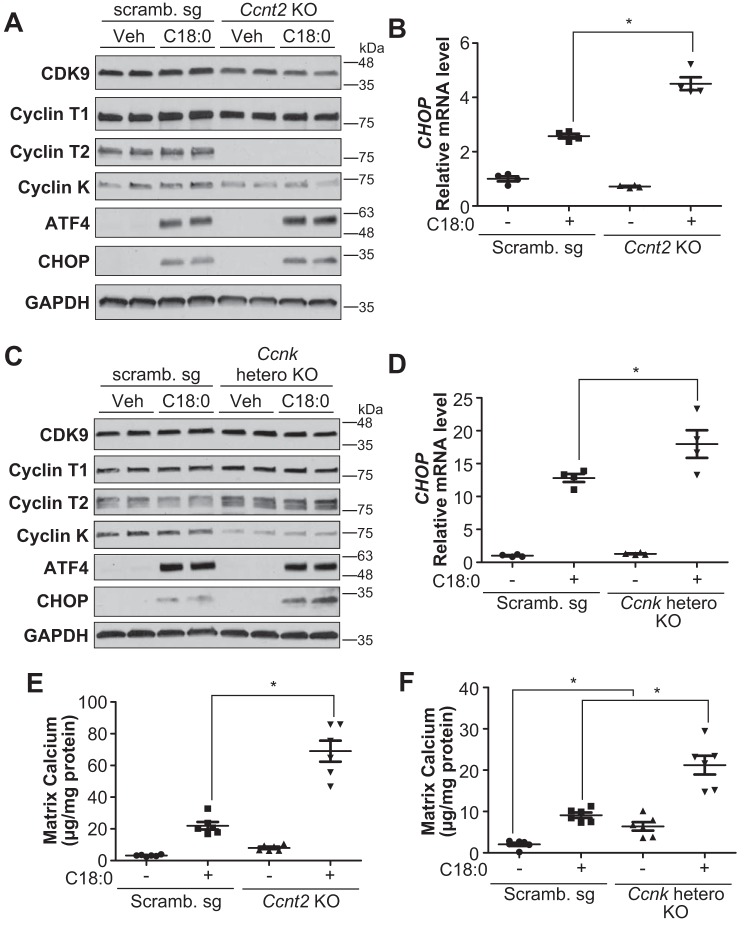
**Cyclin T2 (*Ccnt2*) and cyclin K (*Ccnk*) deficiency induces CHOP expression and calcification, opposite of cyclin T1 deficiency.** *A,* immunoblot analysis of CDK9, cyclin T1, cyclin T2, cyclin K, ATF4, CHOP, and GAPDH proteins in BSA (*Veh*) or 500 μm C18:0 treated scrambled (*Scramb.*) sg or *Ccnt2* KO VSMCs for 6 h. *B,* qRT-PCR analysis of CHOP in BSA (*Veh*) or 250 μm C18:0 treated Scramb. sg or *Ccnt2* KO VSMCs (*n* = 4). *C,* immunoblot analysis of CDK9, cyclin T1, cyclin T2, cyclin K, ATF4, CHOP, and GAPDH proteins in BSA (*Veh*) or 500 μm C18:0 treated Scramb. sg or *Ccnk* heterozygous KO VSMCs for 6 h. *D,* qRT-PCR analysis of CHOP in BSA (*Veh*) or 250 μm C18:0 treated Scramb. sg or *Ccnk* heterozygous KO VSMCs (*n* = 4). *E,* mineralization of *Ccnt2* KO VSMCs. VSMCs were incubated with 2.0 mm phosphate with or without 250 μm C18:0 for 7 days (*n* = 6). *F,* mineralization of *Ccnk* hetero-KO VSMCs. VSMCs were incubated with 2.0 mm phosphate with or without 250 μm C18:0 for 7 days (*n* = 6). One-way ANOVA with a Student-Newman post hoc test was used for statistical analysis. *, *p* < 0.05.

**Figure 7.**
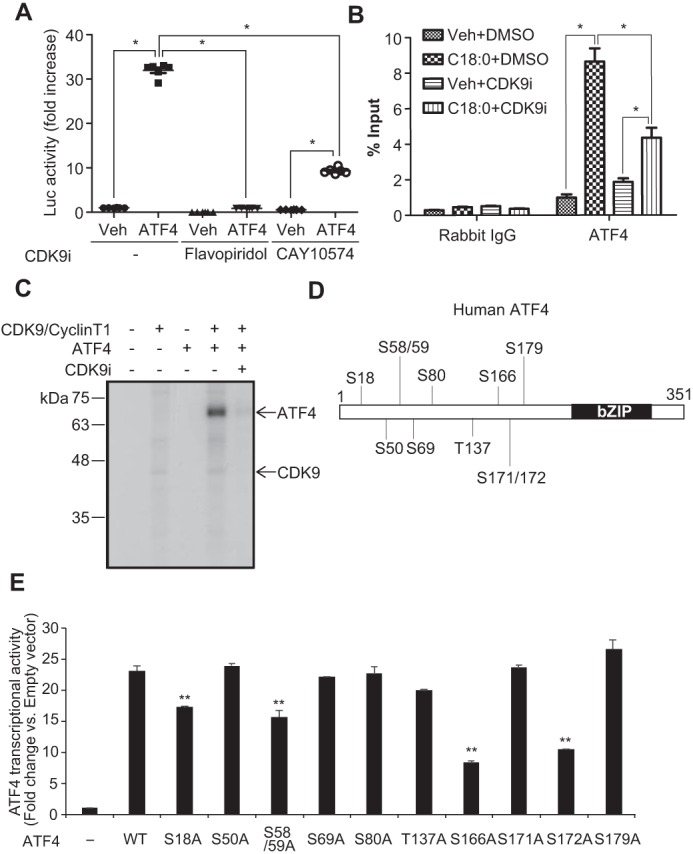
**CDK9–cyclin T1 complex regulates the recruitment of ATF4 to the CHOP promoter under ER stress.** *A,* HEK293T cells were co-transfected with firefly luciferase reporter plasmid containing three repeats of the C/EBP–ATF composite (−303/−292) of the mouse CHOP gene, pCMV-LacZ, and control or human ATF4 expression plasmid. 4 h after transfection; cells were incubated in DMEM containing 10% fetal bovine serum with DMSO (*Veh*) or CDK9 inhibitors (*CDK9i*) such as 300 nm flavopiridol or 30 μm CAY10574 for 24 h. The results are expressed as luciferase (*Luc*)/β-gal units of induction (*n*-fold) over the control value for each construct. One-way ANOVA with a Student-Newman post hoc test was used for comparison between vehicle, flavopiridol, and CAY10574-treated cells (*n* = 6). *B,* ChIP analysis of SFA-induced ATF4 recruitment to the C/EBP-ATF composite of the CHOP promoter. VSMCs were pretreated with 10 μm flavopiridol (CDK9i) for 2 h, and then treated with BSA (*Veh*) or 250 μm C18:0 with or without 10 μm CDK9i. After 6 h, chromatin fractions of VSMCs were prepared. ChIP was performed with rabbit control IgG or ATF4 antibodies. Purified immunoprecipitated DNA was analyzed by qRT-PCR with primers for the C/EBP–ATF composite of the CHOP promoter. The results are expressed as the percentage of antibody binding *versus* the amount of PCR product obtained using a standardized aliquot of input chromatin (% input) (*n* = 3). One-way ANOVA with a Student-Newman post hoc test was used for statistical analysis. *, *p* < 0.05. *C,* phosphorylation of ATF4 by the CDK9–cyclin T1 complex was analyzed using an *in vitro* kinase assay. Recombinant human ATF4 and human CDK9–cyclin T1 complex were incubated with or without 100 nm flavopiridol (CDK9i) in a kinase buffer containing [γ-^32^P]ATP. ATF4 was phosphorylated by both CDK9–cyclin T1 and CDK9–cyclin T2 complexes, and flavopiridol blocked phosphorylation of ATF4. The CDK9–cyclin T1 complex more potently phosphorylated ATF4. Additionally, CDK9 itself was autophosphorylated by the CDK9–cyclin T1 complex. *D,* human ATF4 phosphorylation site by CDK9. *E,* luciferase assay using ATF4 with alanine point mutations on each predicted CDK9-specific phosphorylation site. HEK293T cells were co-transfected with firefly luciferase reporter plasmid containing three repeats of the ATF4RE (−303/−292) of the mouse CHOP gene, pCMV-LacZ, and control or human ATF4 WT or serine/threonine mutation expression plasmid (*n* = 3). One-way ANOVA with a Student-Newman post hoc test were used for statistical analysis. **, *p* < 0.01 *versus* ATF4 WT.

The CDK9–cyclin T1 complex is necessary for SFA-induced CHOP expression and vascular calcification by inhibiting ATF4 recruitment onto ATF4RE of the CHOP promoter. Because 1) CDK9-specific inhibition by chemical inhibition and the gene-editing technique was toxic to VSMCs and not suitable for studying its role in the regulation of vascular mineralization (data not shown), and because 2) CDK9 forms three complexes with cyclin T1, cyclin T2, and cyclin K, we hypothesized that each CDK9–cyclin complex might play a distinct role in ER stress-mediated CHOP expression and vascular calcification. As shown in Fig. 5, we successfully generated cyclin T1 (*Ccnt1*) KO VSMCs using the Cas9–CRISPR technique. *Ccnt1* deficiency reduced CDK9 expression and induced cyclin T2 (*Ccnt2*) expression. *Ccnk* expression was not changed in *Ccnt1* KO VSMCs. More importantly, *Ccnt1* deficiency completely blocked C18:0-mediated CHOP expression, despite no alteration in upstream ER stress effectors such as ATF4 (Fig. 5, *A* and *B*). In addition, *Ccnt1* deficiency reduced the induction of CHOP through other ER stress inducers such as thapsigargin and tunicamycin (Fig. 5*C*). Furthermore, *Ccnt1* deficiency significantly attenuated C18:0-induced mineralization of VSMCs (Fig. 5*D*). As shown Fig. 6, we next modulated *Ccnt2* and *Ccnk* in VSMCs by the Cas9–CRISPR technique. We generated *Ccnt2* KO VSMCs and *Ccnk* heterozygous KO VSMCs, because *Ccnk* homozygous KO VSMCs were completely lethal. In contrast to *Ccnt1* deficiency, *Ccnt2* and *Ccnk* deficiencies significantly intensified C18:0-mediated CHOP expression, whereas ATF4 expression was not altered (Fig. 6, *A–D*). Consistent with CHOP induction, both *Ccnt2* and *Ccnk* deficiency augmented C18:0-mediated vascular calcification (Fig. 6, *E* and *F*). *Ccnk* deficiency induced mineralization of VSMCs even in the absence of C18:0 (Fig. 6*F*). Because 1) CDK9 modulation did not affect the expression of ATF4 and 2) ATF4 is the most critical regulator of CHOP expression under ER stress, we determined whether CDK9 affects the recruitment of ATF4 onto the ATF4RE of the CHOP promoter. To specifically study ATF4 recruitment, ATF4RE triplets (3× ATF4RE) were inserted to a luciferase reporter where the luciferase expression is driven on its own through the thymidine kinase promoter (42). ATF4 expression led to >30-fold induction of luciferase activity with 3× ATF4RE (Fig. 7*A*). ATF4-mediated induction of luciferase activity was remarkably lowered with the treatment of CDK9 inhibitors such as flavopiridol and CAY10574 (Fig. 7*A*). To determine that CDK9 regulates the recruitment of ATF4 to the endogenous native CHOP promoter in VSMCs under ER stress, a ChIP assay was performed in VSMCs treated with C18:0 in the presence of CDK9 inhibitors (flavopiridol and CAY10574) (Fig. 7*B*). C18:0 treatment increased the recruitment of ATF4 to the CHOP promoter by 8.3-fold, which was significantly reduced by co-treatment with CDK9 inhibitors (Fig. 7*B*). We next examined whether CDK9, cyclin T1, or other pTEFb components such as RNA polymerase II and BRD4 make a complex with ATF4 using co-immunoprecipitation, GST pulldown, and double-ChIP assays. However, none of these experiments supported the idea that CDK9, cyclin T1, and other pTEFb components physically bind to ATF4 (data not shown). Because CKD9–cyclin T1 is a serine/threonine kinase, we therefore examined whether the CDK9–cyclin T1 complex simply phosphorylates and activates ATF4. As shown in Fig. 7, *C* and *D*, and Table S2, the kinase assay and LC-MS/MS–based proteomics assay revealed that the CDK9–cyclin T1 complex phosphorylated multiple serine/threonine residues of ATF4, including serine 18, serine 50, serine 58, serine 59, serine 69, serine 80, threonine 137, serine 166, serine 171, serine 172, and serine 179, which were completely blocked by CDK9 inhibitor treatment (Fig. 7*C*). To determine which CDK9-mediated serine/threonine phosphorylation is crucial for the transcriptional activity of ATF4, we replaced the serine/threonine with alanine and analyzed the transcriptional activity. Multiple serine/threonine to alanine mutations of ATF4 including S18A, S58/59A, S166A, and S172A significantly reduced the transcriptional activity of ATF4 (Fig. 7*E*).

## Discussion

Recent studies revealed that ER stress pathways contribute to cardiovascular calcification in CKD and atherosclerosis (2–11). We previously reported the following: 1) CKD increases the level of circulating C18:0 through a klotho-dependent mechanism; 2) CKD induces levels of aortic transcriptional ER stress effectors such as ATF4 and CHOP; 3) accumulation of C18:0 induces ER stress, resulting in severe medial calcification *in vivo*; and 4) global and SMC-specific modulations of ATF4 affect medial calcification in CKD (3, 8–11). However, which ATF4 target promotes the development of CKD-dependent medial calcification is not fully understood. In this study, VSMC-specific and sole induction of CHOP, which is a major downstream target of ATF4, caused and enhanced vascular calcification in CKD. The overexpression of CHOP recapitulated the CKD state and induced medial calcification in the absence of renal dysfunction. The results demonstrate that the induction of CHOP by ATF4 is a key event in the pathogenesis of CKD-dependent medial calcification.

CHOP is a strong mediator of apoptosis under ER stress (4, 12, 18). A number of studies have suggested that VSMC apoptosis links vascular calcification and osteogenic differentiation (4, 6, 43–47). Our previous study showed that CKD simultaneously induced vascular apoptosis and ER stress (3, 9, 11). This study confirmed that CHOP overexpression solely induced VSMC apoptosis *in vivo*, accompanied by the induction of CKD-dependent medial calcification. We previously reported that CHOP deficiency/knockdown inhibited vascular apoptosis and atherosclerotic calcification *in vitro* and *in vivo* (9). These results demonstrate that ER stress-mediated VSMC apoptosis plays a critical role in the pathogenesis of CKD-dependent medial calcification.

We also identified new effectors of ER stress, the CDK9–cyclin complexes. Inhibition of CDK9 by chemical inhibition and Cas9–CRISPR gene editing specifically blocked CHOP induction by C18:0. Our results suggest that CDK9 specifically regulates ER stress-mediated CHOP induction but not other ER stress markers. We also demonstrated that CDK9 regulates ATF4 recruitment onto the ATF4RE of the CHOP promoter without affecting the expression of ATF4. We strived to find a physical interaction between P-TEF components (CDK9, cyclin T1, BRD4, and RNA polymerase II) and ATF4 using a number of techniques, including pulldown, double-ChIP, and co-immunoprecipitation (data not shown). Unlike a previous study on amino acid deficiency (48), any physical interactions between ATF4 and P-TEF components were not observed. We demonstrated that the CDK9–cyclin T1 complex was directly activated through phosphorylation at multiple serine and threonine residues, which were blocked by the CDK9 inhibitor. We also demonstrated that multiple phosphorylations of serine/threonine by CDK9 are critical for inducing CHOP. Interestingly, the CDK9–cyclin T1 and CDK9–cyclin T2/K complexes play distinct roles on ER stress-mediated CHOP expression. Cyclin T1 knockout reduced CHOP expression, whereas cyclin T2 and cyclin K knockout augmented CHOP expression induced by ER stress. In addition, cyclin T2–CDK9 blocked phosphorylation of ATF4 by the cyclin T1–CDK9 complex (data not shown). These data suggest that cyclin T1 works as an endogenous activator on ER stress-mediated CHOP expression and vascular apoptosis, whereas cyclin T2 and cyclin K work as endogenous inhibitors by blocking ATF4 recruitment onto the CHOP promoter.

In this study, we focused on examining the role of CDK9 in regulating ER stress-mediated CHOP expression. In addition to CDK9, however, our inhibitor library screening found that five inhibitors for the phosphoinositide 3-kinase (PI3K)-mechanistic target of rapamycin (mTOR) pathway blocked C18:0-mediated CHOP expression (Table 1). The data suggest that PI3K–mTOR signaling contributes to ER stress-mediated CHOP expression and vascular calcification. Several *in vitro* studies have shown that the mTOR inhibitor rapamycin inhibits mineralization and osteogenic differentiation of VSMCs (49, 50), suggesting that mTOR may play a causative role in the development of vascular calcification. Taken together, the mechanism by which mTOR inhibition blocks vascular mineralization may be through the attenuation of ER stress-mediated CHOP expression. Further mechanistic and *in vivo* studies will be required to determine the following: 1) how mTOR inhibition blocks ER stress-mediated CHOP expression, and 2) which mTOR contributes to vascular calcification *in vivo*.

**Figure 8.**
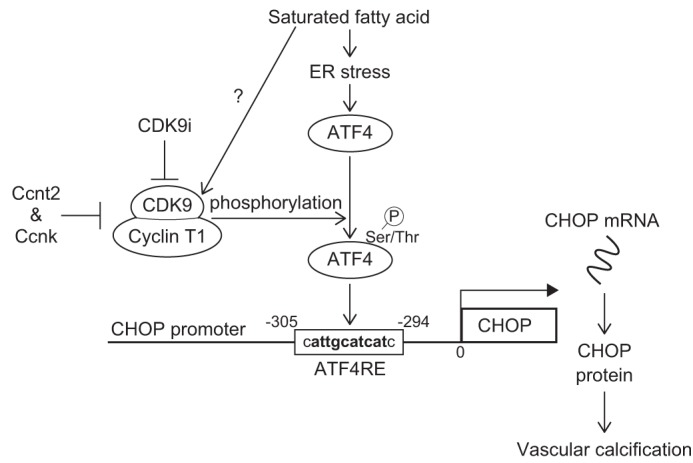
**CDK9–cyclin T1 complex activates ATF4 activity through multiple phosphorylations under ER stress, leading to vascular calcification in CKD.** The proposed mechanism of CHOP induction by ATF4 with CDK9–cyclin complexes in vascular calcification is shown. CKD increases levels of C18:0. C18:0 activates the PERK–eIF2α–ATF4–CHOP axis of ER stress signaling, resulting in calcification. The CDK9–cyclin T1 complex phosphorylates and mediates translocation of ATF4 to the ATF4RE of the CHOP promoter, and cyclins T2/K compete with cyclin T1 for binding with CDK9.

Taken together with previous reports from our group and other groups, this study reveals that ER stress-mediated CHOP is a critical event in the pathogenesis of vascular calcification. The CDK9–cyclin T1 complex is an essential component in ER stress-mediated pro-apoptotic CHOP expression and vascular calcification by phosphorylation-mediated ATF4 activation. In addition, cyclin T2 and cyclin K endogenously inhibit CHOP induction by competitively binding to CDK9 (Fig. 8). Moving toward translational medical inhibition of the cyclin T1/CDK9–CHOP pathway may be a potential strategy to treat CKD-dependent vascular calcification.

## Experimental procedures

### Animals

DBA/2J mice were purchased from The Jackson Laboratory. SMMHC–Cre^ER(T2)^ mice were generated as described previously (3, 11, 51, 52). For SMC-specific CHOPTG mice, we introduced the transgene at the Rosa26 locus by the recombinase-mediated cassette exchange method that we recently developed to circumvent the inherent problem of random insertion via traditional pronuclear injection. In brief, we transfected 5 × 10^6^ R26FNF3–1F1 ES cells with 15 μg of pFLSLF3-HA CHOP and 15 μg of pCAG-Flpe (Addgene plasmid 13787). From the 96 clones tested, we found seven clones with the expected genotype, which were also G418-sensitive, indicating successful exchange of the original neo cassette for the transgene cassette at the Rosa26 locus. Karyotypically normal ES clones were microinjected into C57BL/6 blastocysts to produce chimeric founders at the Bioengineering Core Facility, University of Colorado. The generated mice were named Rosa26–CHOP conditional TG (Rosa26–CHOP^loxtg/+^) mice. All of the mouse strains were backcrossed more than 10 times with C57Bl/6J mice. The genetic backgrounds were checked at the BioResources Core Facility of the Barbara Davis Center, University of Colorado-Denver. The Rosa26–CHOP^loxtg/+^ mice were intercrossed with SMMHC–Cre^ER(T2)^ mice to obtain SMMHC–Cre^ER(T2)^;CHOP^loxTG/+^ mice. Because the SMMHC–Cre^ER(T2)^ transgene was inserted on the Y chromosome, only males were used in this study. The 5-week-old males were intraperitoneally injected with either 1 mg of tamoxifen in vegetable oil or vehicle for 5 consecutive days to produce SMC-specific CHOPTG mice and control mice, respectively. After the injections, the mice were maintained on a special diet (TD10364) for 12 weeks. CKD was induced using 5/6 nephrectomy as reported previously, whereas sham operation was used as an NKD condition (8, 9, 53). VSMCs, skin fibroblasts, peritoneal macrophages, neutrophils, and hepatocytes were isolated as described previously. For the studies with the CDK9 inhibitor, 8-week-old DBA2J males were subjected to either sham operation or 5/6 nephrectomy. Two weeks after the surgeries, mice were treated by daily i.p. injections of either vehicle (5% DMSO/PBS) or 0.5 or 2.5 mg/kg flavopiridol for 8 weeks (54, 55). Animal experiments were approved by the Institutional Animal Care and Research Advisory Committee, University of Colorado at Denver.

### Cell cultures

Human VSMCs were purchased from Applied Biological Materials (T0515, Richmond, British Columbia, Canada). A mouse VSMC line (MOVAS-1) was provided by Mansoor Husan at the University of Toronto (Toronto, Canada) (3, 9, 10). VSMCs were maintained in DMEM containing 10% fetal bovine serum with 100 units/ml penicillin and 100 μg/ml streptomycin. For calcification assays, MOVAS cells were treated with BSA or 250 μm C18:0 with 2.6 mm P_i_ every 2 days for a week.

### Preparation of lentiviral particles and CRISPR–Cas9 system-based gene knockout of Ccnt1, Ccnt2, and CcnK genes

Scrambled (Applied Biological Materials Inc., Richmond, British Columbia, Canada) or target gene sgRNA (listed in Table S1) was inserted in lenti-CRISPR version 2 plasmid (Addgene) or pLenti-U6-sgRNA-SFFV-Cas9–2A-Puro plasmid (Applied Biological Materials Inc), including the Cas9 coding sequence in the lentiviral sequence region. HEK293T cells were seeded at a density of 6 × 10^5^ cells/well in 6-well plates, grown overnight, and then transfected with 300 ng of psPAX2, 100 ng of pMD2, and 400 ng of each sgRNA CRISPR–Cas9 lentivirus plasmid (plasmid amount rate 3:1:4) using Turbofect (ThermoFisher Scientific). Media were changed to new media, and media containing lentiviral particles were collected 2 days later. Lentiviral media were centrifuged once at 1500 × *g* for 3 min, and the supernatant was collected. MOVAS cells were seeded in 6-well plates and infected 24 h later with each lentiviral media in the presence of 10 μg/ml Polybrene. Cells were treated with 5 μg/ml puromycin for selection of infected cells. Total RNA of heterogeneous cells was collected, and cDNA synthesis was conducted from the RNA template, followed by high-resolution melting analysis with a StepOne Plus qPCR instrument (Applied Biosystems) to check for mutations occurring on regions around each sgRNA target sequence (primer sequences listed in Table S1) (56). Heterogeneous cells with gene mutations were plated at 0.5 cells/well in a 96-well plate to obtain a single cell clone. Next, total RNA of each single cell-derived clone was collected to check for mutations using high-resolution melting qRT-PCR, and then each mutated sequence was analyzed by DNA sequencing (Eurofins Genomics Company). Protein from gene-edited clones was prepared and analyzed by immunoblot analysis to determine whether gene knockout was complete.

### RNA analysis

qRT-PCR assays were performed using an Applied Biosystems StepOne Plus qPCR instrument. Quantitative expression values were calculated by the absolute standard curve method using plasmid template (Open Biosystems) containing each target gene cDNA. Primer sequences that are fully validated were obtained from the Primer bank (Harvard University, https://pga.mgh.harvard.edu/primerbank/)^3^ and published previously (3, 11).

### Transfection and luciferase assays

HEK293T cells were plated at a density of 1 × 10^5^ cells/well in 24-well plates and grown overnight. Cells were transfected with 200 ng of firefly luciferase reporter plasmids (pGL3, Promega), 50 ng of expression plasmid (FLAG-Atf4-pcDNA3.1), and 100 ng of pCMV-LacZ vector using Turbofect (ThermoFisher Scientific). After 24 h of transfection, cells were harvested with 100 μl of passive lysis buffer, and luciferase activities were measured using a luciferase assay system (Promega). Firefly luciferase activity was divided by β-gal activity to obtain a normalized value, the relative luciferase unit.

### Immunoblot analysis

Cell and tissue lysates were prepared using RIPA buffer (150 mm NaCl, 1% Nonidet P-40, 0.5% sodium deoxycholate, 0.1% SDS, 50 mm Tris, pH 8.0). Cells were disrupted by pipetting 10–15 times and centrifuged at 13,800 × *g* for 10 min at 4 °C, and the supernatant was collected as total cell lysates. The samples were separated by SDS-PAGE, transferred to a nitrocellulose membrane, and immunoblotted with the following antibodies: ATF4 (D4B8), CHOP (D46F1), ATF3(D2Y5W), BiP (C50B12), Bcl-2 (D17C4), and cyclin T1 (D1B6G) from Cell Signaling Technology; CDK9 (D-7), cyclin K (G-11), Bax (B-9), GADD34 (H-193), β-Actin (C-4), and GAPDH (V-18) from Santa Cruz Biotechnology; and cyclin T2 (NBP1-87592) from Novus Biologicals. Samples were visualized using horseradish peroxidase coupled to appropriate secondary antibodies with enhancement by an ECL detection kit (ThermoFisher Scientific).

### Kinase inhibitor library screening

The kinase inhibitor library was purchased from Cayman Chemicals. Human VSMCs (Applied Biological Materials) were seeded in 6-well plates and grown overnight. Following pretreatment with 10 μm of each drug for 2 h, VSMCs were co-treated with 250 μm 18:0 and 10 μm of each drug for 6 h. VSMCs lysates were then prepared using RIPA buffer. CHOP band intensity was semi-quantified using ImageJ and corrected by actin band intensity. The inhibitors found to be effective in inhibiting SFA-induced CHOP protein expression are listed in Table 1.

### Histological analysis

Calcified lesions in the aortic arches were analyzed as described previously (3, 9, 11, 53). To distinguish between calcified lesions and pigments on the aortic valve leaflets, histological images were captured before and after von Kossa staining. Apoptotic cells in the medial layer of aortic arches were detected using an In Situ Cell Death Detection Kit (Roche Applied Science), as described previously (8, 9, 11, 57). At least five sections from each sample were analyzed.

### Calcium content in cultured cells and aortas

Matrix calcium deposition in cultured cells and aortas was quantified as described previously (3, 8, 9, 11, 53, 57).

### ChIP analysis

The samples were prepared using SimpleChIP® enzymatic chromatin IP kit (agarose beads) from Cell Signaling Technology. ChIP was performed using rabbit IgG control (CST catalog no. 2729) or ATF4 antibody (D4B8) that was incubated for 30 min at 65 °C in ChIP elution buffer. The supernatant was treated with proteinase K for over 2 h at 65 °C and purified using a spin column. Purified immunoprecipitated DNA was analyzed by qRT-PCR. The results are expressed as the percentage of antibody binding *versus* the amount of PCR product obtained using a standardized aliquot of input chromatin (% of input). The primers for the C/EBP-ATF composite region of the mouse CHOP promoter are as follows: sense, 5′-CACCTCCCACCACCATC-3′, and antisense, 5′-GGCTTGAGAGTCTACGTTGT-3′.

### In vitro kinase assay

Recombinant human ATF4 with an N-terminal His_6_–calmodulin tag, active human CDK9–cyclin T1 complex with an N-terminal His_6_ tag, and active human CDK9–cyclin T2 complex with an N-terminal GST tag were purchased from Prospec (Israel), ThermoFisher Scientific, and SignalChem (Richmond, British Columbia, Canada). 10 ng of ATF4 and 10 ng of CDK9–cyclin T1 complex or CDK9–cyclin T2 complex were mixed and incubated in a kinase buffer (70 mm Tris-HCl, 10 mm MgCl_2_, and 5 mm DTT) with 1 μCi of [γ-^32^P]ATP for 40 min at 30 °C. The samples were separated by SDS-PAGE. After electrophoresis, a polyacrylamide gel was dried for 2 h at 80 °C. The dried gel was autoradiographed using X-ray film for 16 h at −80 °C. The phosphorylation sites of ATF4 by CDK9–cyclin T1 *in vitro* kinase assay were determined at the Mass Spectrometry Core facility, University of Colorado, using Agilent 6550 QTOF after trypsin (Preomics), Glu-C (Promega), or Asp-N (Promega) digestion.

### Statistics

Data were collected from more than two independent experiments and are reported as the mean ± S.E. Statistical analysis was performed using a two-tailed Student's *t* test for two group comparisons and a one-way ANOVA with a Student-Newman post hoc test or a two-way ANOVA for multigroup comparison. Significance was accepted at *p* < 0.05.

### Study approval

All animal protocols and experimental procedures were approved by the Institutional Animal Care and Use Committee at the University of Colorado, Denver.

## Author contributions

Y. S. and M. M. conceptualization; Y. S., K. W., X. Z., W. S. C., and M. M. resources; Y. S., K. O., S. K., A. L. K., S. M.-A., and M. M. data curation; Y. S., A. L. K., and M. M. software; Y. S., S. K., A. L. K., S. M.-A., and M. M. formal analysis; Y. S., K. O., A. L. K., and M. M. validation; Y. S., K. O., S. K., A. L. K., S. M.-A., and M. M. investigation; Y. S., K. O., A. L. K., and M. M. visualization; Y.ß., K. O., S. K., S. M.-A., and M. M. methodology; Y. S. and M. M. writing-original draft; Y. S., A. L. K., and M. M. writing-review and editing; M. M. supervision; M. M. funding acquisition; M. M. project administration.

## Supplementary Material

Supporting Information

## Footnotes

3 Please note that the JBC is not responsible for the long-term archiving and maintenance of this site or any other third party hosted site.

## Footnotes

2 The abbreviations used are:

CKD: chronic kidney disease
CDK: cyclin-dependent kinase
ATF4: activating transcription factor 4
CHOP: CCAAT/enhancer-binding protein homologous protein
SFA: saturated fatty acid
VSMC: vascular smooth muscle cell
NKD: normal kidney condition
TG: transgenic
ANOVA: analysis of variance
GAPDH: glyceraldehyde-3-phosphate dehydrogenase
qRT-PCR: quantitative RT-PCR
MAPK: mitogen-activated protein kinase
MEK: mitogen-activated protein kinase/extracellular signal-regulated kinase kinase
PERK: PKR-like endoplasmic reticulum kinase
ER: endoplasmic reticulum
PI3K: phosphatidylinositol 3-kinase
mTOR: mechanistic target of rapamycin
DMEM: Dulbecco's modified Eagle's medium
ES: embryonic stem.
