# Supplementary material for: The CDK9–cyclin T1 complex mediates saturated fatty acid–induced vascular calcification by inducing expression of the transcription factor CHOP
Source: J Biol Chem. 2018 Sep 12;293(44):17008–20. doi: 10.1074/jbc.RA118.004706 (PMC6222109; doi:10.1074/jbc.RA118.004706)
Supplement: Supporting Information [file supp_293_44_17008__index.html]

The CDK 9–cyclin T1 complex mediates saturated fatty acid–induced vascular calcification by inducing expression of the transcription factor CHOP — CDK9-cyclinT1-CHOP in regulating vascular calcification — The CDK9–cyclin T1 complex mediates saturated fatty acid–induced vascular calcification by inducing expression of the transcription factor CHOP — CDK9–cyclin T1–CHOP in regulation of vascular calcification — Supporting Information 

# The CDK9–cyclin T1 complex mediates saturated fatty acid–induced vascular calcification by inducing expression of the transcription factor CHOP

## Supporting Information

- Supporting Information (to be published online) - Supplement
