# Supplementary material for: The CDK9–cyclin T1 complex mediates saturated fatty acid–induced vascular calcification by inducing expression of the transcription factor CHOP
Source: J Biol Chem. 2018 Sep 12;293(44):17008–20. doi: 10.1074/jbc.RA118.004706 (PMC6222109; doi:10.1074/jbc.RA118.004706)
Supplement: Supporting Information [file supp_RA118.004706_139186_2_supp_198146_p6n9q7.pdf]

Table S1 used sgRNA and primer sequences in this paper

Gene	sgRNA sequence	Vector	HRM qRT-PCR primer sequence	
Cdk9	CTCACCGTATAACCGCTGCA	lenti CRISPR v2 (Addgene)	CAGAAGGTGGCTCTGAAGAAA	Sense
			CACAGAAGTCAAACACCAGATAGA	Antisense
Ccnt1 (Cyclin T1)	GACGTTGTCCCATGTCCTGG	pLenti-U6-sgRNA-SFFV-Cas9-2A-Puro	ATGGAGGGAGAGAGGAAGAA	Sense
		(abm Inc.)	TGATCGTCAGTTGTGAGACG	Antisense
Ccnt2 (Ctclin T2)	CTGCGATTGTTTATATGCAC	lenti CRISPR v2 (Addgene)	CGTCTCAATGTCTCTCAGCTTAC	Sense
			GAAGACAAGCGTGTGCTACT	Antisense
Ccnk (Cyclin K)	AATAAATGATTCCAGTTGCC	lenti CRISPR v2 (Addgene)	TACTGGGATAAGAAGGACCTAGC	Sense
			GGGAATTGTTTGAAGGAATGGAAC	Antisense

Table S2. Results from the proteomics analysis

enzyme	SM Score	SPI%	Mod Site	Sequence
Glu-C	16.34	90.6	S18s	EVLVGDLMsPFDQSGLGAEES
Glu-C	15.99	90.7	T137t	ETNKQPPQtVNPIGHLPES
Glu-C	17.61	93.3	M4m M17m S18s	EmSFLSSEVLVGDLmsPFDQSGLGAEES
Trypsin	17.99	86.3	S69s	KAGSSEWLAVDGLVsPSNNSKE
ASP-N	17.48	94.3	S69s	SDKAKAGSSEWLAVDGLVsPSNNSKED
ASP-N	12.89	65	S50s	LDDYLEVAKHFKPHGFsSD
ASP-N	20.48	91.9	S69s N72n	VDGLVsPSnNSKED
Trypsin	10.01	69.1	S80s	(K)EDAFsGTDWMLEKM
ASP-N	20.66	96.7	S59s S69s	SDKAKAGSsEWLAVDGLVsPSNNSKED
ASP-N	14.4	69.3	S58s S69s	SDKAKAGsSEWLAVDGLVsPSNNSKED
Glu-C	15.29	94.2	S179s	ESLTKPDQVAPFTFLQPLPLSPGVLSSTPDHSFsLEL
Glu-C	19.71	88.3	S172s	ESLTKPDQVAPFTFLQPLPLSPGVLSsTPDHSFSLEL
Glu-C	16.82	87.2	S166s S171s	ESLTKPDQVAPFTFLQPLPLsPGVLsSTPDHSFSLEL
